# Designing Mindfulness Conversational Agents for People With Early-Stage Dementia and Their Caregivers: Thematic Analysis of Expert and User Perspectives

**DOI:** 10.2196/40360

**Published:** 2022-12-06

**Authors:** Cassandra E L Seah, Zheyuan Zhang, Sijin Sun, Esther Wiskerke, Sarah Daniels, Talya Porat, Rafael A Calvo

**Affiliations:** 1 Dyson School of Design Engineering Imperial College London London United Kingdom; 2 Ladywell Dementia Team Lewisham Council London United Kingdom; 3 Care Research & Technology Centre UK Dementia Research Institute London United Kingdom

**Keywords:** mindfulness, dyadic, dementia, caregivers, user needs, intervention, mindfulness, user, feedback, design, accessibility, relationships, mindset, essential

## Abstract

**Background:**

The number of people with dementia is expected to grow worldwide. Among the ways to support both persons with early-stage dementia and their caregivers (dyads), researchers are studying mindfulness interventions. However, few studies have explored technology-enhanced mindfulness interventions for dyads and the needs of persons with dementia and their caregivers.

**Objective:**

The main aim of this study was to elicit essential needs from people with dementia, their caregivers, dementia experts, and mindfulness experts to identify themes that can be used in the design of mindfulness conversational agents for dyads.

**Methods:**

Semistructured interviews were conducted with 5 dementia experts, 5 mindfulness experts, 5 people with early-stage dementia, and 5 dementia caregivers. Interviews were transcribed and coded on NVivo (QSR International) before themes were identified through a bottom-up inductive approach.

**Results:**

The results revealed that dyadic mindfulness is preferred and that implementation formats such as conversational agents have potential. A total of 5 common themes were also identified from expert and user feedback, which should be used to design mindfulness conversational agents for persons with dementia and their caregivers. The 5 themes included enhancing accessibility, cultivating positivity, providing simplified tangible and thought-based activities, encouraging a mindful mindset shift, and enhancing relationships.

**Conclusions:**

In essence, this research concluded with 5 themes that mindfulness conversational agents could be designed based on to meet the needs of persons with dementia and their caregivers.

## Introduction

### Background

Dementia has been predicted to affect an estimated 78 million people by 2030 [1]; therefore, new interventions are needed to support caregivers and persons with dementia (dyads). Mindfulness-based interventions (MBIs) are an example receiving increasing attention. MBIs for participants with dementia have been shown to improve quality of life [2,3] and decrease depressive symptoms [2]. MBIs have also played a role in improving self-reported stress, depression, anxiety, burden, quality of life, cognition, and mood in caregivers of persons with dementia [4-14].

Few studies of MBIs use a dyadic approach in which both the person with dementia and their caregiver engage together in a mindfulness activity [15-19]. Dyadic MBI approaches have shown benefits, such as improved well-being, quality of life, depressive symptoms, relaxation, awareness, acceptance, and resilience [15,16,18]. Although studies reveal positive benefits for dyadic MBIs, there is a lack of focus on how the interventions are designed for the stakeholders. Studies often adapted mindfulness interventions without stating the specific adaptations or the reasons why they were made [3].

Most dyadic MBI studies were performed in physical group settings [5,15-17], with one study conducting home sessions using recordings [20] and another using guided telephone calls [4]. Introducing more advanced technologies such as conversational agents to dyadic mindfulness for persons with dementia and their caregivers is a novel approach and not previously studied. Conversational agents refer to artificial intelligence or computer programs that use natural language processing to converse with people [21]. The use of conversational agents could improve accessibility, as they allow users to access them in the comfort of their own homes instead of traveling to a physical location. They also allow users to access them at any time, as opposed to having to wait for a guide through physical groups or telephone calls. Conversational agents may also support individuals with limited digital competencies, which may be beneficial not only for older adults but also for people with cognitive challenges [22], offering lower barriers to use by using voice as a communication medium, as opposed to using a graphical interface device. Users simply need to speak to conversational agents instead of learning how to navigate through digital interfaces, thereby enhancing accessibility. Conversational agents may also provide more personalization and guidance as compared with recordings, by guiding dyads step by step in a personalized manner, as opposed to a static video or voice recording. They may also provide more scalability as compared with physical groups or guided telephone calls, as they do not require a mindfulness expert to be present each time, allowing for automation as opposed to a manual approach. Conversational agents could benefit current mindfulness interventions for dyads by improving accessibility, scalability, guidance, and personalization, thus offering opportunities for mindfulness to be seamlessly integrated into the dyads’ lives. To understand this, research on conversational agents for persons with dementia, which was relatively scant [6], was explored, with some interesting use cases such as dementia detection [7]. Unfortunately, conversational agents for dementia are inadequately discussed in the scientific community, with studies lacking rigor [6]. However, a recent study revealed that persons with dementia can use embodied conversational agents independently in their home environment [8]. This showed good engagement with the system, revealing the potential for conversational agents to meet the basic and higher-level needs of people living with dementia [8]. To the best of our knowledge, this is the first study of dyadic MBIs supported by technologies such as conversational agents.

Dyadic mindfulness conversational agents may be a more useful way to engage persons with dementia and their caregivers. Although dyadic mindfulness has shown benefits for persons with dementia and their caregivers, physical group settings and guided telephone calls were used, which were not scalable, as they required a trainer for each session, and were not accessible, as users could not use them whenever they wanted. Physical group settings were also not accessible as they required users to travel to a physical location. Recorded formats provide for accessibility and scalability needs, given that users know how to navigate the digital space, but are not personalized and guided such as physical group sessions or telephone calls. A way to bridge this gap is to use conversational agents for dyadic mindfulness, providing accessibility and scalability while also ensuring guidance and personalization for users. However, for future studies to design and test the effectiveness of dyadic mindfulness conversational agents, the needs and preferences of persons with dementia and their caregivers need to be understood first.

To understand the design recommendations, we first examined the existing literature. The recommendations below only address designing dyadic MBIs or conversational agents for persons with dementia. Few recommendations could be found, as studies primarily focused on the effectiveness of dyadic MBIs or conversational agents rather than on the design of the interventions. Recommendations for designing dyadic mindfulness programs for persons with dementia and their caregivers included the following: first, allowing separate interventions for each individual on different occasions [3]. Care should be taken when providing interventions with a dyadic approach because caregivers may be reluctant to fully focus on themselves, as they are concerned about their partners. They may also not feel comfortable discussing their concerns. Second, mindfulness guides must be able to manage participants’ negative emotions [20]. Mindfulness interventions may provide a mental space for negative emotions to arise, and trainers need to be able to guide participants through the process. Third, the needs of caregivers should be met [3]. It was important to ensure that the needs of caregivers were taken care of, as they may be more worried about the well-being of the persons with dementia than about their own well-being. On the other hand, recommendations for designing conversational agents for persons with dementia included improving the quality of speech recognition [8]. Automatic speech recognition quality and synthesis were technical problems that required improvement because they negatively impacted the adaptiveness and usability of conversational agents. Recommendations from the existing literature only addressed parts of the proposed intervention—dyadic mindfulness or conversational agents for persons with dementia. Recommendations for dyadic MBIs using conversational agents for persons with dementia and their caregivers could not be found. Therefore, this study’s research aims involved understanding the dyadic mindfulness conversational agent needs of persons with dementia and their caregivers. This study sought to address this aim through feedback from both the expert and user perspectives. This will enable future designers and developers to create appropriate dyadic mindfulness conversational agents based on user needs.

### Research Question

What is important when designing mindfulness conversational agents for persons with dementia and their caregivers?

## Methods

### Ethical Considerations

Ethics approval was granted for this study by Imperial College Research Ethics Committee (21IC6573). Informed consent was obtained from all the participants before they participated in the study.

### Study Design

Semistructured interviews were conducted via Microsoft Teams. The first author interviewed 5 dementia experts, 5 mindfulness experts, 5 persons with early-stage dementia, and 5 dementia caregivers individually based on a predefined topic guide and through a 1-hour interview. Dementia and mindfulness experts were recruited between March and June 2021. Persons with dementia and their caregivers were recruited between October and November 2021. Multimedia Appendix 1 outlines the structure that was adopted for the interviews with experts and users.

### Participants

Individuals with expertise in mindfulness training or dementia were recruited from research networks of coinvestigators using an opportunity sampling method. Persons with dementia and caregivers were recruited through dementia groups on the social media platform Facebook (Meta Platforms Inc), where a volunteer sampling approach was used. Persons with early-stage dementia and caregivers individually responded to advertisements on various dementia groups. As it was particularly challenging to recruit individuals who were either caregivers to persons with dementia or persons with early-stage dementia, the caregivers and persons with dementia who participated in this study were given GBP £20 (US $23.60) and GBP £40 (US $47.10), respectively, as an appreciation for their time. Interviewees were informed that they could withdraw at any time without giving a reason. All the participants had to be aged ≥18 years, able to use Microsoft Teams, and communicate in English. In addition, the following criteria had to be met:

- Dementia experts had to be service providers in health care facilities working with and planning programs for persons with dementia.
- Mindfulness experts had to be teachers and practitioners of mindfulness methodologies or therapies, such as Mindfulness-Based Cognitive Therapy or Mindfulness-Based Stress Reduction or mindfulness activities such as meditation without a clinical focus or equivalent.
- Persons with dementia were in the early stage of the illness and able to consent for themselves.
- Caregivers had to be primary caregivers of an individual with a dementia diagnosis (early stage of any type) and must have provided care for at least 3 months before recruitment.

### Data Collection and Analysis

The sessions were recorded using Microsoft Teams before they were transcribed. The transcripts were analyzed using NVivo (QSR International; version 12). A thematic analysis approach was used to identify, analyze, and report patterns within the data [9]. Thematic analysis is a flexible approach that organizes and describes data sets that are rich in detail, allowing for the interpretation of various aspects of the research topic [9]. This approach was used because it enabled the identification of important themes across expert and user feedback.

For expert feedback, an inductive bottom-up approach was used by 2 researchers independently. Data were coded as the researchers read through the data. Codes were created and applied, identifying emerging topics as the data were analyzed. Pattern coding was then performed to condense the data into fewer analytical concepts. Themes were subsequently identified from the pattern codes and compared by the 2 researchers across different experts’ data. Themes were chosen based on the following: (1) whether they were identified by both researchers and both types of experts, and (2) if they were not identified by both researchers, they should have had a significant number of codes from expert feedback that both researchers subsequently explored and agreed to include. Cohen κ coefficient [10], was calculated to measure the level of agreement between the 2 researchers. Calculations using the formula reflected a value of 0.69, showing moderate agreement between researchers for expert feedback [11].

For user feedback, attribute codes were first developed based on the main topics from the interview questions, organizing data by (1) living situation, (2) socioeconomic factors, (3) health factors and care needs, (4) coping methods, (5) hobbies and daily life, (6) main challenges, (7) use of technology, (8) use of mindfulness, (9) factors to consider for design, and (10) individual activity preferences. After this, 2 researchers, CELS and ZZ, separately used an inductive method—an open coding approach—before pattern coding was performed to condense the codes. Themes were subsequently identified from the pattern codes. The 2 researchers then came together to compare the themes, where both researchers reached a consensus about which themes to include after discussion and justification. Themes were chosen based on the following: (1) whether they were identified by both researchers and both types of users, and (2) if they were not identified by both researchers, they should have had a significant number of codes from user feedback that both researchers subsequently explored and agreed to include. Calculations using the Cohen κ coefficient reflected a value of 0.77, showing moderate agreement between the researchers for user feedback [11].

Representative data consisting of expert and user quotes were included to support the themes identified. A cross-comparison analysis across themes from expert and user perspectives was performed to identify similarities. The analysis was reviewed by 4 other researchers. Data and all appropriate documents will be stored for a minimum of 10 years after the study is completed. All video recordings were transcribed in a timely fashion and removed from Microsoft Teams.

## Results

### Participants

A total of 5 dementia experts and 5 mindfulness experts who fulfilled the inclusion criteria completed the interviews, and all 10 interviews were analyzed. Most of the dementia experts (3/5, 60%) interviewed were facilitators at day activity centers, and all of them (5/5, 100%) were female. All the mindfulness experts (5/5, 100%) were mindfulness teachers, and most mindfulness experts (3/5, 60%) were male.

In all, 5 caregivers and 7 persons with early-stage dementia responded to the advertisements posted on Facebook dementia groups; 2 persons with dementia were excluded because they had difficulty understanding questions or experienced challenges using Microsoft Teams. The 10 users completed the interviews, and all 10 interviews were analyzed. A total of 60% (3/5) of caregivers cared for parents with dementia, whereas 40% (2/5) of caregivers were spouses of persons with dementia. Most caregivers (4/5, 80%) were female. Of the 5 people with early-stage dementia who were interviewed, 4 (80%) were supported by their spouses. They were able to manage daily tasks with some difficulty but had assistance from others. However, 20% (1/5) of persons with dementia lived independently in a retirement village, with assistance from paid caregivers who visited occasionally. Most persons with dementia (3/5, 60%) were female. Table 1 presents more information on the participants.

**Table 1:** Participant details (N=20).

Type of participant			Value, n (%)
**Dementia expert (n=5)**			
	Nursing home allied health professional	1 (20)	
	Hospital staff	1 (20)	
	Day activity center facilitator	3 (60)	
**Mindfulness expert (n=5)**			
	Mindfulness meditation teacher	1 (20)	
	Mindfulness movement teacher	1 (20)	
	Mindfulness teacher	3 (60)	
**Persons with dementia (n=5)**			
	Person with early-stage vascular dementia	1 (20)	
	Person with early-stage dementia	1 (20)	
	Person with early-stage frontotemporal dementia	3 (60)	
**Caregivers (n=5)**			
	Carer for person with early-stage vascular dementia	1 (20)	
	Carer for person with early-stage frontotemporal dementia	1 (20)	
	Carer with experience caring for person with early-stage dementia	3 (60)	

### Preferences for Conversational Agents That Support Dyadic Mindfulness

Most participants, all (5/5, 100%) caregivers and 60% (3/5) of persons with dementia, had past experiences using various forms of mindfulness practices. Although not all users had experiences with mindfulness, all 10 (5 caregivers and 5 persons with dementia) participants mentioned that they would use mindfulness practices after trying a mindful exercise. This indicated that mindfulness was appealing to the participants. With regard to practicing mindfulness as a pair, 80% (4/5) of the caregivers and 60% (3/5) of the persons with dementia expressed interest. However, 20% (1/5) of caregivers and 40% (2/5) of persons with dementia preferred to engage in mindfulness individually. According to most users, dyadic mindfulness was preferred.

Most participants, 60% (3/5) of caregivers and 80% (4/5) of persons with dementia, were familiar with using conversational agents. They used them to contact people, monitor the home, plan things using the calendar, set reminders and alarms, have conversations, tell the weather, and play music. They had past experiences with various types of conversational agents—Alexa, Siri, and Google Assistant. Caregivers and persons with dementia were asked to speculate on format preferences (conversational agents or videoconferencing software) to practice dyadic mindfulness; 60% (3/5) of caregivers and 60% (3/5) of persons with dementia preferred using conversational agents. Conversational agents were preferred, as they were something that the users could do on demand instead of having to arrange and schedule a zoom session. The users mentioned that conversational agents can be accessed whenever they wanted and that it did not matter whether it was at night. However, 40% (2/5) of caregivers and 40% (2/5) of persons with dementia preferred using videoconferencing owing to a more personal touch. Overall, mindfulness interventions designed for dyads could incorporate the use of conversational agents, given the majority’s familiarity with the technology and their likelihood of using it. However, the interviewees were tech-savvy and may not be representative of the target population. It may be helpful for future studies to include participants who are not tech-savvy to understand their perspectives as well.

### Designing for Dyadic Mindfulness Conversational Agents—Common Expert and User Themes

#### Overview

A total of 9 expert themes and 7 user themes were identified from the inductive thematic analysis of expert and user interviews to identify what was necessary in designing mindfulness conversational agents for persons with dementia and caregivers. The expert and user themes identified are listed accordingly in Textbox 1, where 5 common themes were identified.

**Comparison of expert and user themes.**

**Expert themes**

- Embracing ability
- Evoking the 5 senses
- Fostering engagement
- Creating habits

**User themes**

- Alleviate stress
- Alleviate worries

**Common themes**

- Enhancing accessibility
- Encouraging mindful mindset shift
- Enhancing relationships
- Cultivating positivity
- Providing simplified tangible and thought-based activities

#### Enhancing Accessibility

First, 60% (3/5) of dementia experts, 60% (3/5) of mindfulness experts, all (5/5, 100%) caregivers, and 80% (4/5) of persons with dementia emphasized the importance of keeping solutions designed for persons with dementia and their caregivers as accessible as possible, with activities explained in a simple and straightforward manner. It was necessary to avoid complex sentences, as they may cause difficulties in following instructions according to person with dementia D5. Second, providing guidance may also help facilitate accessibility, according to 40% (2/5) of dementia experts, 80% (4/5) of mindfulness experts, and 20% (1/5) of persons with dementia. Having expert guidance could help participants to have clarity and offer more support for people who need it, especially for those who may be progressively getting worse. Third, it would also be essential to consider how the activities designed for the dyad progress as the condition of the person with dementia declines, ensuring that users would still be able to use solutions with ease, as mentioned by 20% (1/5) of dementia experts, 80% (4/5) of caregivers, and all (5/5, 100%) persons with dementia. This could be accomplished by breaking down the tasks or simplifying programs for persons at different stages of dementia.

And it has to be as simple as possible, even for early stage, you know, as simple as possible.

Dementia expert D4

So your version of simple and what actually works for me can be very, very different.

Person with dementia D1

#### Encouraging Mindful Mindset Shift

Participants in the 4 groups believed that a mindful mindset shift may be able to benefit the dyad through present-moment awareness and having no attachments or aversions to experiences. Present-moment activities were used and preferred by 80% (4/5) of dementia experts, all (5/5, 100%) mindfulness experts, all (5/5, 100%) caregivers, and all (5/5, 100%) persons with dementia to enhance present-moment awareness. Worries that come with a progressing illness could be mitigated through the effects of present-moment activities, where the hustle and bustle stop for a moment and there is a sense of calm, changing the relationship with negative experiences to one of ease rather than one of struggle. In addition, 80% (4/5) of mindfulness experts and 40% (2/5) of persons with dementia identified the need to have no attachment or aversion to events, learn to have acceptance, and be comfortable with discomfort. Where pleasant or unpleasant feelings arise, thoughts should be allowed to come and go while being mindful—not reacting to or judging them. This means that the participants would be comfortable with discomfort because a “different way of being is actually okay.” Moreover, 40% (2/5) of persons with dementia exemplified this by accepting and acknowledging uncomfortable experiences and waiting patiently for negative emotions to pass. Interventions for dyads should encourage a mindset shift, allowing dyads to stay in the present moment and learn to have no attachment or aversion to experiences.

Being in the moment is just so important, isn’t it, for everybody, really, but I think specifically for someone who lives with an illness (dementia) and something which is obviously progressing...

Dementia expert D1

If I’m upset, I just acknowledge that I’m upset and I have a reason to be upset and wait till it passes, really.

Person with dementia D5

#### Enhancing Relationships

Social connectedness was widely identified as a factor that should be incorporated into dyad activities by 80% (4/5) of dementia experts, all (5/5, 100%) mindfulness experts, all (5/5, 100%) caregivers, and 80% (4/5) of persons with dementia. In particular, social connectedness between the dyads was important according to mindfulness experts M2 and M5. This was further emphasized by 60% (3/5) of caregivers and 40% (2/5) of persons with dementia who mentioned having a worsened relationship because of dementia needs and symptoms. Caregivers found it difficult to live with the person with dementia, grappling with the “huge personality change.” Similarly, 40% (2/5) of persons with dementia expressed negative changes in their relationships with their loved ones. As the condition of persons with dementia declines, they lose their abilities and have changes in their personalities, resulting in worsened relationships. It would be important for interventions to improve social connectedness between the dyads. Dyadic dynamics needs to be carefully designed to encourage a stronger bond between the pair, as mentioned by mindfulness experts M4 and M5.

Quality of the relationship between caregiver and the person with dementia is vital.

Mindfulness expert M2

My husband says you have become a liability to me and that really, really hurt because we had been a team.

Person with dementia D1

#### Cultivating Positivity

A total of 40% (2/5) of dementia experts, all (5/5, 100%) mindfulness experts, 80% (4/5) of caregivers, and 40% (2/5) of persons with dementia identified the need to focus on the positive. They recommended activities that provided calm, appreciation, and loving kindness to enable the dyads to cultivate positivity. First, activities promoting calmness in the dyads were identified as essential by 40% (2/5) of dementia experts and 40% (2/5) of mindfulness experts. This resulted in a sense of peace, which was positive. Second, all (5/5, 100%) mindfulness experts, 40% (2/5) of caregivers, and 40% (2/5) of persons with dementia recommended activities that had elements of appreciation. Appreciating the “duality of human nature” and embracing situations in their entirety according to mindfulness expert M2 could be a way to bring some positivity to the lives of the dyads. Moreover, 40% (2/5) other caregivers mentioned noticing and appreciating the little moments of positivity instead of ruminating or dwelling on negative thoughts. In addition, 40% (2/5) of persons with dementia had a positive outlook on life despite having dementia and suggested that it was important to see the positive in the negative and learn to still be joyful and appreciative in the face of adversity. According to the experts and users, encouraging appreciation is essential for cultivating positivity in the dyads. Finally, 40% (2/5) of mindfulness experts recommended activities that had elements of loving kindness. Practicing loving kindness may also help to cultivate positivity. In essence, elements of positivity were recommended to be incorporated into interventions designed for dyads, enabling them to better cope with the challenges that come with dementia.

What I would really recommend building in would be something on using mindfulness in terms of appreciation and in terms of gratitude, so I think this sort of practices can be really helpful for people.

Mindfulness expert M5

I’ve re-evaluated and rediscovered there’s a joy and a lightness in this new as well. I’ve looked death in the face and decided that I can dance and do and stand.

Person with dementia D1

#### Providing Simplified Tangible and Thought-Based Activities

According to experts and users, dyad activities could be tangible or thought based. In all, 60% (3/5) of dementia experts and 80% (4/5) of mindfulness experts recommended using mindful breathing, where the focus is on noticing the breath, as it is tangible and easy to grasp, which may be particularly useful for dyads. All (5/5, 100%) caregivers and all (5/5, 100%) persons with dementia would similarly do tangible activities like deep breathing, as it was a tangible and effective way to calm them down. In all, 20% (1/5) of mindfulness experts, 60% (3/5) of caregivers, and 60% (3/5) of persons with dementia would recommend or do other forms of tangible activities such as body scan meditation, where the focus is on the sensations one feels in the body. However, 40% (2/5) of caregivers and 40% (2/5) of persons with dementia mentioned not wanting to do body scan meditation, citing reasons of not understanding how to do the activity, lack of interest, and potential of feeling overwhelmed.

A total of 40% (2/5) of dementia experts and 60% (3/5) of mindfulness experts conducted thought-based activities that used reflection to help dyads generate insights into self. Mindfulness expert M4 explained that through mindful reflection, one would be aware of their thoughts, feelings, and sensations, enabling them to prevent automatic rumination of thoughts and breaking the cycle of how fear-based thoughts affect unhealthy behavior. In addition, all (5/5, 100%) caregivers and 80% (4/5) of persons with dementia would do thought-based activities, focusing on reflection through dyadic gratefulness, where one reflects on positive things with a partner. Moreover, 80% (4/5) of caregivers and 60% (3/5) of persons with dementia were open to other thought-based activities such as letting go, which helps users to be comfortable with discomfort and feel less overwhelmed. The activity encourages being open and accepting to difficult emotions and experiences, rationalizing the process using metaphors. However, 40% (2/5) of persons with dementia had difficulties understanding the abstract components that had metaphors. Overall, certain simplified tangible and thought-based activities may be suitable for dyads, depending on their individual needs.

#### Comparison With Prior Work

When compared with design recommendations identified in the literature—(1) dyadic mindfulness programs for persons with dementia and their caregivers as well as (2) conversational agents for persons with dementia—most of the themes uncovered were novel and reflected the needs from a holistic perspective, based on user and expert feedback. The existing design recommendations may have been created from the researchers’ perspectives, which may explain why most of them did not coincide with the user and expert themes, as seen in Table 2.

**Table 2:** Comparison of the dyadic mindfulness conversational agent design needs highlighted by users and experts with the existing recommendations from the literature.

Expert themes			User themes		Design recommendation (literature)
**Common themes**					
	Enhancing accessibility	Enhancing accessibility		Quality of speech recognition needs to be improved [8]	
	Encouraging mindful mindset shift	Encouraging mindful mindset shift		N/A^a^	
	Enhancing relationships	Enhancing relationships		N/A	
	Cultivating positivity	Cultivating positivity		N/A	
	Providing simplified tangible and thought-based activities	Providing simplified tangible and thought-based activities		N/A	
Embracing ability			N/A		N/A
Evoking the 5 senses			N/A		N/A
Fostering engagement			N/A		N/A
Creating habits			N/A		N/A
N/A			Alleviate stress		N/A
N/A			Alleviate worries		N/A
N/A			N/A		Allow separate interventions on different occasions [3]
N/A			N/A		Mindfulness guides need to be able to manage participants’ negative emotions [20]
N/A			N/A		Ensure that the needs of caregivers are met [3]

^a^N/A: not applicable.

### Designing for Dyadic Mindfulness Conversational Agents—Expert Themes That Were Not Present in User Themes

A total of 4 themes were identified by experts and were not present in the user-identified themes. These include embracing ability, evoking the 5 senses, fostering engagement, and creating habits.

#### Embracing Ability

A total of 60% (3/5) of dementia experts and 20% (1/5) of mindfulness experts highlighted the importance of seeing dyads under a different light, focusing on abilities. They thought that after a diagnosis, dyads may focus on the negative aspects of dementia, and learning to see themselves and each other under a different lens could be useful. Similar sentiments were noted in the subthemes that identified role reversal, autonomy, and empowerment of dyads as important. Role reversal was brought up by 40% (2/5) of dementia experts and 20% (1/5) of mindfulness experts, where modifying the role for the persons with dementia could better engage them, preventing passivity. Role reversal was also used to help caregivers step out of their caring role to take a break. Second, encouraging autonomy was essential, and according to 40% (2/5) of dementia experts, it could come in the form of providing options for choices to be made by dyads. Third, according to 60% (3/5) of dementia experts, empowering dyads can help them to see that, although they are affected by the disease, there are many things that they can still do.

Actually he did tell us as well, that he started to see her in a different light...

Dementia expert D1

#### Evoking the 5 Senses

In all, all (5/5, 100%) dementia experts and 80% (4/5) of mindfulness experts mentioned the use of activities that stimulated the 5 senses. Activities used by dementia and mindfulness experts had an emphasis on the different senses, allowing for nonverbal communication needs to be met. Although it may be beneficial for activities designed for dyads to incorporate the 5 senses, care must be taken to prevent overloading their sensory capabilities.

We just use everything...all the senses and the garden and nature...

Dementia expert D5

#### Fostering Engagement

According to 5 (100%) dementia experts, activities have to be engaging. It is important to create a positive association with the activity. Moreover, 60% (3/5) of dementia experts identified providing variety as an important component for engaging the users. Dementia experts D1, D3, and D4 provided a wide scope of activities with varying difficulties, providing flexibility for the users to choose what they preferred. Providing choices of format, frequency, and duration may also be important for fostering engagement.

It needs to be fun.

Dementia expert D2

#### Creating Habits

Mindful habits can be created by incorporating mindfulness into current routines and using reminders to aid the practice. According to 40% (2/5) of dementia experts and 60% (3/5) of mindfulness experts, activities could be woven into existing routines to introduce new habits, rather than having them as separate activities. Reminders on how to perform mindfulness activities, on what the activities comprise, and to do the activities could also help to facilitate the formation of mindful habits, as mentioned by 40% (2/5) of dementia experts.

What is their routine and then build it into that, the activities list rather than separate because separate will never happen...

Dementia expert D3

### Designing for Dyadic Mindfulness Conversational Agents—User Themes That Were Not Present in Expert Themes

A total of 2 themes were identified by users and were not present in the expert-identified themes. These include alleviating stress and alleviating worries.

#### Alleviate Stress

Caregivers and persons with dementia are highly stressed, and interventions should address this need. A total of 60% (3/5) of caregivers had other caregiving duties in addition to caring for persons with dementia, and 40% (2/5) of caregivers also had other personal illnesses. These factors may have contributed to why 80% (4/5) of the caregivers felt highly stressed and unable to cope. In addition, 80% (4/5) of persons with dementia experienced high levels of stress because of their dementia symptoms, 2 of which also had other accompanying illnesses, adding on to their stress levels. Most caregivers and persons with dementia experienced high levels of stress, and it is necessary for interventions designed for dyads to lower stress levels.

It does sometimes get completely overwhelming that you just can’t deal with anymore, you know. And I have felt I suppose over the last year I felt waves of being more overwhelmed, getting closer to tears or something more quickly than I have in the past.

Caregiver C2

#### Alleviate Worries

Caregivers and persons with dementia had high levels of worries because of dementia, and interventions should address this issue. A total of 80% (4/5) of caregivers mentioned struggling with worries they had about the person they care for, revealing an inability to cope with the situation. In addition, all (5/5, 100%) persons with dementia mentioned losing their abilities as one of the main challenges they faced, causing them to feel worried. These challenges reflected high levels of worries, and it is important for interventions to ensure that dyads are better able to manage their worries.

Mostly I worry about him.

Caregiver C5

## Discussion

### Principal Findings

A total of 5 themes outlining the user needs for designing dyadic mindfulness conversational agents were gathered from expert and user perspectives in this study. These themes could be used to design future dyadic mindfulness conversational agents.

Studies on dyadic mindfulness [15,16,18] and conversational agents for persons with dementia [23,24] were relatively scant, with a focus on assessing effectiveness rather than on the design and user experience of the intervention. Only 4 user needs were gathered from past studies on dyadic mindfulness and those on conversational agents for dementia, and it was unclear whether the needs were derived based on user feedback. Furthermore, there were no studies on dyadic mindfulness conversational agents; therefore, user needs could not be found. This revealed the lack of understanding of needs from the users’ perspective, which is what this study sought to address. The resulting themes, derived from an inductive process, provided interesting insights that helped meet the study’s objective—to fill the gap in the literature concerning the design needs of persons with dementia and their caregivers for a dyadic mindfulness conversational agent approach from expert and user perspectives.

The 5 common themes identified from expert and user feedback inform the design of mindfulness conversational agents for dyads. These themes incorporated the voices of dementia experts, mindfulness experts, persons with dementia, and caregivers. The value of approaches incorporating user opinions has been amply demonstrated in the literature, notably in the field of designing for persons with dementia and caregivers [21,25]. Gathering feedback from a holistic range of stakeholders had several advantages, including knowledge cocreation across dementia expertise, mindfulness expertise, and lived experiences. In this process, inclusivity and feasibility were enhanced by integrating user and expert perspectives, as they provided an insight into important considerations that no single stakeholder group could have identified alone.

From the 5 common themes, we noted a few important considerations. First, enhancing accessibility for both persons with dementia and caregivers is essential. This can be achieved by ensuring that the activities are simple and straightforward, where guidance is provided. Ensuring that they would still be able to perform the intervention as the condition of the person with dementia deteriorates should also be considered. However, what may be simple for designers may not be simple for persons with dementia, so there is a need to test and iterate the design of mindfulness conversational agents with dyads. Care must be taken to ensure that activities can be used by persons with dementia, even as their condition declines. Providing tangible activities that are less focused on cognitive capabilities could enable dyads to stay engaged as their condition deteriorates. Furthermore, to enhance accessibility, by providing guidance, technological implementation formats could be used for dyadic mindfulness. However, dyads may possess a wide spectrum of technological capabilities. For older adults who lack the high comfort and digital literacy skills of digital-native generations [12], it may be useful to provide more accessible and user-friendly technologies. This could be implemented using conversational agents, as users need not learn how to navigate a digital interface and simply have to talk to the conversational agent, which would guide them through an activity. There may be a learning curve to use conversational agents as well, but having a dyadic arrangement would be helpful as caregivers would be able to guide persons with dementia if needed. However, activities should be designed such that persons with dementia would be able to use them independently, as the activities should also benefit the caregiver, instead of having them worry about the person with dementia. This can be done by considering the difficulty of the activities and the phrasing of words used to convey the activities and ensuring that there is enough time for dyads to process and respond.

Second, encouraging a mindful mindset shift involving (1) staying in the present moment and (2) having no attachment or aversion to experiences is recommended. Present-moment activities were used by almost all the experts and users and should be incorporated into mindfulness interventions for dyads. Although having no attachment or aversions to experiences was identified by only 2 groups—80% (4/5) of mindfulness experts and 40% (2/5) of persons with dementia—it may be useful for dyads as 80% (4/5) of caregivers expressed negative situations where they felt like they were unable to cope, and no attachment and aversion in these situations could help them to manage. Similarly, persons with dementia D1, D3, D4, and D5 mentioned concerns about not being able to do things they were able to do before, and it may be essential to alleviate worries by promoting no attachment and aversion to their current situation. Interventions designed for dyads to promote no attachment and aversion, helping them to manage negative situations by learning to be comfortable with discomfort, may be a useful tool to cope as the condition of the person with dementia declines. Although both mindful mindset shifts may be useful, it may be easier to stay in the present moment with tangible-based activities such as deep breathing, which is easy to follow and focus on throughout an activity session. Having no attachment or aversion to experiences requires self-control, as it is not a typical response to experiences. When implementing activities of both kinds, designers must ensure that the activities are accessible. In addition, to embody a mindful mindset shift, where one lives in the present moment and has no attachment or aversion to experiences, extensive practice and habit formation are required. This could be facilitated through a technological approach such as conversational agents, where daily reminders and guided practices could aid habit formation, leading to a mindful mindset shift.

Third, there is a need to enhance relationships, as social connectedness was brought up by almost all the experts and users as essential. This may be particularly important between dyads, as the participants mentioned having worsened relationships because of dementia needs and symptoms. Most caregivers interviewed cared for parents with dementia, whereas most persons with dementia interviewed had spouses who cared for them. Whether it was a spousal relationship or child-parent relationship, persons with dementia and their caregivers had worsened relationships, which a dyadic mindfulness conversational agent could potentially address. In a previous study, dyadic mindfulness was facilitated by having one partner share whatever came to their mind and another partner listen to the other’s contemplation, which increased social connectedness [13]. Dyadic interaction could be further explored and integrated, for example, where dyads share with each other about something that they are grateful to each other for. However, it is important to note that this would encompass creating interventions for 2 different individuals and care must be taken to ensure that both their needs can be met. Although dyadic mindfulness conversational agents may provide opportunities to enhance relationships, dyadic arrangements may also prevent individuals from sharing their true concerns. For instance, mindfulness experts M4 and M5 mentioned that caregivers may not be willing to share the exasperation they feel from caregiving and persons with dementia may not be willing to share the fears they experience with dementia. Thought needs to be given on how to navigate both paths to manage the pros and cons of dyadic arrangements. Allowing separate interventions on different occasions, as suggested by the existing literature, may be able to mitigate this. Similarly, although dyadic mindfulness user preferences revealed that most users, 4 caregivers and 2 persons with dementia (6/10, 60%) would use mindfulness as a pair, 40% (4/10) of users, 1 caregiver and 3 persons with dementia, would prefer to do mindfulness individually.

Fourth, cultivating positivity is important and can be accomplished by providing calm, appreciation, and loving kindness. However, according to the 7 attitudes of mindfulness [14], cultivating positivity is not one of the attitudinal foundations of mindfulness practice. Nevertheless, dementia experts, mindfulness experts, persons with dementia, and caregivers mentioned the need to cultivate positivity, and it should still be incorporated into mindfulness interventions for dyads. It is also important to note that cultivating positivity does not necessarily mean to remove negativity, as mindfulness also promotes learning to be comfortable with discomfort. Instead, mindfulness for dyads should provide a balance of activities that cultivates a mindful mindset shift while also incorporating elements of positivity, as dyads face high levels of stress and worries with the diagnosis.

Finally, although it was recommended by both experts and users that tangible activities be provided, not all tangible activities are straightforward. Mindful breathing, a tangible activity, was easy to grasp and favored by majority of experts as well as all users. By contrast, body scan meditation was tangible, but not as straightforward. Some users mentioned feeling overwhelmed when practicing body scan meditation, whereas others did not know how to perform the activity. For example, when caregiver C2 tried body scan meditation, she mentioned feeling overwhelmed by the existing pain she felt from her body. “By focusing on pain...it was amplifying it,” making her “realise how bad it is and how widespread it is” when she focused on her body. Different activities may elicit different responses from the users, with some unexpected and unintended consequences. It may be important to provide choices of tangible activities from which the users may choose or provide alternative paths within the activity itself so that the users have alternatives, should a path not work well for them. It is also essential to note that although tangible activities, according to experts, may be easier for users to grasp, certain tangible activities could not be understood by persons with dementia. When abstract components and multiple concepts were introduced to persons with dementia, 40% (2/5) of them were unable to understand the activity. Even though tangible activities may be easier to grasp, the complexity of the activity and the phrasing used to explain each activity should be simplified, in particular, to cater to the needs of persons with dementia. Similarly, for thought-based activities, simplified versions were also recommended, as metaphors used to explain concepts were too difficult for 40% (2/5) of persons with dementia to follow. Care must be taken to ensure that the users are not overloaded with cognitive tasks that are too challenging.

By comparing and identifying the common themes mentioned by the 4 groups—dementia experts, mindfulness experts, persons with dementia, and caregivers—this study provided a comprehensive view of the themes needed to design dyadic mindfulness conversational agents. Most of the existing guidelines from the literature did not coincide with the common themes highlighted by experts and users.

### Conversational Agent Implementation Method

Dyadic MBIs have been implemented in physical group settings, home settings through recordings, and guided telephone calls [15-19] but have yet to be implemented using more accessible formats and more advanced technologies such as conversational agents. Current implementation methods such as physical groups have accessibility limitations, as they require dyads to travel to a physical location, limiting the participants who are not able to be physically present. Situations such as the ongoing COVID-19 pandemic also make it difficult for people to attend in-person interventions. Physical groups and guided telephone calls also have scalability limitations and limited access, as they require a facilitator to be present, which limits the number of people who can access the intervention at each point in time. The users would also have to schedule a meeting to practice mindfulness but are not able to do it whenever they need it. By contrast, recordings of mindfulness allow for greater scalability and accessibility, as it can be used anywhere with 24/7 access, given that the users have adequate digital literacy, but do not allow for a personalized or guided experience, which physical group settings and guided telephone calls provide.

Using conversational agents may be able to address these needs while also providing for accessibility and scalability needs. Mindfulness conducted through conversational agents would be available to access in the users’ homes 24/7, not requiring them to travel to a physical location and allowing them to access it whenever needed. Conversational agents are also more accessible in terms of digital literacy needs, allowing dyads to simply speak to the conversational agent. The technology is scalable, allowing many people to simultaneously access mindfulness activities. Furthermore, it is also able to provide personalized and guided mindfulness activities, which recordings are not able to do.

This study revealed that dyadic mindfulness was of interest, and using novel implementation formats such as conversational agents, ideally one with a more personal touch, was preferred by most users. It was particularly interesting for conversational agents to be preferred by the users, as they were generally older. However, it is important to note that the users interviewed were able to navigate the internet space, use software such as Microsoft Teams independently, and have strong digital literacy skills, which may not be representative of the entire population. Moreover, 60% (3/5) of caregivers and 80% (4/5) of persons with dementia also had prior experience using conversational agents.

### Strengths and Limitations

This study had multiple strengths, where it provided an understanding of the preferences and needs of persons with dementia and their caregivers with regard to dyadic mindfulness conversational agents. The insights gathered were novel, as dyadic mindfulness conversational agents had not been created before, and, therefore, the needs and preferences were not known. In addition, using both expert and user perspectives strengthened the study, providing insights from lived-experience pairs, as well as experts who had planned and executed dementia and mindfulness programs. However, there were a few limitations.

First, the results may not be representative of the target population, as persons with dementia and caregivers who volunteered to participate in the study, as well as mindfulness and dementia experts who were identified, were tech-savvy and able to navigate videoconferencing tools and social media. As the interviews were conducted digitally because of the COVID-19 pandemic, only interviewees who had adequate competence and confidence in using the digital tools were recruited for the study, limiting the generalizability of the results. As there would likely be interviewees who are not as technologically savvy, this resulted in the study’s sample being biased. It may be beneficial to include a more comprehensive range of users in future studies.

Second, as interviewees had no prior experience with mindfulness conversational agents for dyads, it was difficult to determine their needs when designing such interventions. To mitigate this, dyadic mindfulness conversational agents were explained to the participants and we had to rely on their knowledge and past experiences with conversational agents, dementia, and mindfulness programs. Consequently, the responses from the users did not have a strong emphasis on conversational agent–specific feedback. This could be because the users did not have a prototype to experiment with and provided feedback based on their past experiences. Future research should use prototypes of mindfulness conversational agents for dyads to experience before obtaining their feedback. This would ensure that their needs are appropriately identified.

Third, it was challenging to recruit persons with dementia and caregivers for this study. Attempts to mitigate this were made by recruitment through multiple research networks and social network groups. This resulted in a small sample size of participants who volunteered to participate, which may have affected the validity of the study. Nevertheless, the richness of insight resulting from the thematic analysis of the interviews was satisfactory for the purpose of the study. For future studies, larger sample sizes should be recruited.

Fourth, there may have been a volunteer bias, as the persons with dementia and caregivers in this study volunteered to participate through the advertisements posted. They may not represent the general population that includes less empowered or motivated persons and, therefore, would affect the validity of the study. It may be useful for future studies to increase the number of volunteers to prevent volunteer bias.

### Conclusions

This study helped fill the gap in the literature concerning the needs of persons with dementia and their caregivers for dyadic mindfulness conversational agents. The results of the semistructured interviews suggested that dyadic mindfulness for persons with dementia and their caregivers was preferred, with potential implementation formats such as conversational agents. Using technologies such as conversational agents could potentially enhance the accessibility, scalability, personalization, and guidance of dyadic mindfulness interventions. These interventions may be particularly important during circumstances such as the COVID-19 pandemic where digital technologies could enable mindfulness services to be provided to dyads. This study also revealed the needs to be considered when designing dyadic mindfulness conversational agents. A total of 5 themes were identified through expert and user interviews. The five themes, defined through an inductive process, included (1) enhancing accessibility, (2) cultivating positivity, (3) providing simplified tangible and thought-based activities, (4) encouraging mindful mindset shift, and (5) enhancing relationships. The development of mindfulness conversational agents for dyads should follow recommendations from both expert and user perspectives to ensure that dyadic needs can be met.

## Appendix

Multimedia Appendix 1 Expert and user topic guide focus.

## Abbreviations

MBI: mindfulness-based intervention

## Data Availability

The data sets generated and analyzed during this study are available from the corresponding author upon reasonable request. Please send an email to CELS.
