# Supplementary material for: Designing Mindfulness Conversational Agents for People With Early-Stage Dementia and Their Caregivers: Thematic Analysis of Expert and User Perspectives
Source: JMIR Aging. 2022 Dec 6;5(4):e40360. doi: 10.2196/40360 (PMC9768661; doi:10.2196/40360)
Supplement: Multimedia Appendix 1 [file aging_v5i4e40360_app1.pdf]

Expert and user topic guide focus

Activity/ Method	Min	Description	Materials	Outcomes
Welcoming	2	Welcome participants and thank them for taking part in the study<br><br>Explain what would be explored in the interview	-	• Familiarise participants with interview aims
Understanding Current Users, Context and Methods	10	Ask about dyads' living situation, socio-economic factors, care needs, hobbies and daily life<br><br>Ask participants about current challenges dyads face<br><br>Ask participants what approaches they used that may be useful for dyads, exploring why they work	Interview questions	• Understand dyads<br>• Understand current challenges faced by dyads<br>• Understand current methods used and mechanisms of practices that may work well for dyads
Explaining and Understanding Mindfulness Practices	10	Introduce mindfulness to participants<br><br>Ask participants about mindfulness preferences	-	• Experts and users understand mindfulness practices<br>• Understand mindfulness preferences
Understanding Dyadic Mindfulness Conversational Agent Preferences	10	Ask participants about their preferences with regard to dyadic mindfulness conversational agents	Interview questions	• Understand dyadic mindfulness conversational agent preferences
Understanding Needs of Designing Mindfulness Conversational Agents for Dyads	25	Ask participants about the needs of mindfulness conversational agents for persons with dementia and caregivers with regard to explaining the intervention, ensuring intervention is suitable for dyads, executing the intervention, ensuring engagement of intervention, format of intervention as well as social component of intervention	Interview questions	• Understand mindfulness conversational agent needs for persons with dementia and caregivers

Closing	3	Thank participants for taking part in the study		• Conclude interview

This is a Multimedia Appendix to a full manuscript published in the J Med Internet Res.
For full copyright and citation information see http://dx.doi.org/10.2196/jmir.xxxx
